# Bibliometric Analysis of the Current Landscape of Global Scientific Production on the Development of Vaccines against Dental Caries

**DOI:** 10.1155/2022/7678891

**Published:** 2022-05-02

**Authors:** Alejandra Torres-Loyola, Carlos Rojas-Arana, Arnaldo Munive-Degregori, Maria Eugenia Guerrero, Franco Mauricio, Josmel Pacheco-Mendoza, Frank Mayta-Tovalino

**Affiliations:** ^1^Medical School, Faculty of Health Sciences, Universidad Científica Del Sur, Lima, Peru; ^2^Department of Master's in Library and Information Science, Faculty of Arts and Human Sciences, Universidad Nacional Mayor de San Marcos, Lima, Peru; ^3^Department of Medico Surgical Stomatology, Faculty of Dentistry, Universidad Nacional Mayor de San Marcos, Lima, Peru; ^4^Department of Postgraduate, Faculty of Dentistry, Universidad Nacional Federico Villarreal, Lima, Peru; ^5^Unidad de Investigación en Bibliometría, Universidad San Ignacio de Loyola, Lima, Peru

## Abstract

**Objective:**

To perform a bibliometric analysis of the scientific research on the development of vaccines against dental caries.

**Methods:**

An extraction of the scientific production published on the development of vaccines against dental caries between 2011 and 2020 was carried out from the Scopus database. Microsoft Excel was used for the elaboration of tables and SciVal for the bibliometric analysis of the data, which were divided into indicators of production, impact, and collaboration. Finally, VOSviewer was used for co-occurrence analysis of keywords and collaborative networks.

**Results:**

106 studies were retrieved from the Scopus database, which were conducted on the development of dental caries vaccines within the years 2011–2020. Wuhan University, in China, was the university with the highest scientific production on the subject, with 4 publications. Regarding the most productive journals, the first place was occupied by the Journal of Dental Research with 7 publications. Regarding the most productive journals, the first place was occupied by the Journal of Dental Research with 7 publications. The highest percentage of the documents analyzed was in quartile 1 journals and in the national collaboration pattern.

**Conclusion:**

Most of the manuscripts regarding the development of vaccines against dental caries were published in China and in *Q*1 quartile journals. In addition, Yan Huimin, Yang Jingyi, Zhou Dihan, Yang Yi, Li Yuhong and Fan Mingwen were found to top the list of most productive authors. The Journal of Dental Research was also identified as the most productive and cited journal.

## 1. Introduction

Bibliometrics is a relevant tool used for the evaluation of academic productivity, scientific articles, teams, and authors themselves, among others, within the framework of scientific research [1]. This type of study has had a great repercussion in recent years due to its valuable usefulness in measuring the impact and influence of the multiple publications in the existing scientific literature [2], thus confirming, through the number of citations granted in particular [3], their contribution and importance in the respective field. Its usefulness lies in measuring the scientific production, turning these elaborations into measurable trend indicators to allow weighting the performance of these [4, 5].

Dental caries continues to reside as one of the most prevalent diseases, with an approximate figure of 2.3 billion people affected worldwide [6], with preschoolers and schoolchildren being the population with the highest incidence, up to 30–60% [7]. Physiologically, it implies a decrease in the amount of minerals in the teeth, due to a constant low pH. Physiologically, it involves a decrease in the amount of minerals in the teeth, due to a constant low pH [8]. The importance lies in the implication of multiple fields such as nutritional, labor, and family, added to the lack of timely treatment can lead to various complications such as abscesses, tooth loss, and osteomyelitis, among others [9].

Vaccines against this pathology are developed mainly with the purpose of providing immunity. To prevent the development and colonization of *Streptococcus mutans*, one of the most frequent etiological germs [10], those that act directly on bacterial proteins, such as the recombinant protein and some adjuvants to improve their immune effect, have been developed and investigated [11]. Others are studying the use of existing vaccines that may influence caries [12].

Because caries can appear at any time of life, a vaccine against caries should provide long-lasting immunity. Therefore, future studies should focus on new antigenic targets to produce vaccines with increasingly effective results. This is a matter of concern, since caries physiologically develops differently from acute infections, and there is a probability that the vaccine will not have the same effectiveness, even though it would have a positive impact on the population at risk [13]. A major limitation for the development of this vaccine is that not many studies have been carried out in humans, since a cross-reaction with heart and skeletal muscle tissue has been described; for this reason, research has continued other types of antibodies that would not have this effect to achieve an effective and safe vaccine in the future [14].

The present bibliometric study will provide a better approach to the subject, encouraging the institutions and/or authors to produce more scientific research, since dental caries generates high health costs for the person who will need the healing service, presenting multiple complications, also considering the uncertainty about the development of a vaccine and considering the lack of bibliometric bibliography [15]. In addition, it will allow us to know what the current scientific production is to observe quantitatively the production and the relevance in the future.

Therefore, the objective of this bibliometric study was to perform an analysis of the global scientific production on the development of vaccines for dental caries, with a search based on the literature present in the Scopus database during 2011–2020.

## 2. Methods

### 2.1. Study Design

A bibliometric study was conducted, which is retrospective, cross-sectional, and descriptive. On September 18, 2021, secondary data published in the Scopus database were evaluated. A total of 536 metadata corresponding to the subject were found.

### 2.2. Database

The Scopus database, which is one of the largest databases worldwide, was used to collect all the metadata that will be considered in the present study, together with the SciVal software, which was used for the subsequent analysis of the metadata.

### 2.3. Search Strategy

An advanced search strategy was used, with variants of the keywords extracted from the Emtree thesaurus of Embase and the Medical Subject Heading (MESH) of PubMed. When using the Scopus database, we limited the search to only manuscripts such as articles, reviews, short surveys, systematic reviews, and clinical trials as our sources. Sources such as conference papers, editorials, book chapters, notes, letters, and erratums were excluded. Results from 2021 and prior to 2011 were also excluded to avoid delays related to indexing in Scopus, due to the requirement of at least 6 months' update according to the metrics. The validity of the search strategy was tested by reviewing the retrieved documents. Since our software (SciVal) can analyze the last decade, the study period was limited from 2011 to 2020. The complex formula used for the search is as follows: TITLE-ABS-KEY (“Caries” OR “caries dental” OR “cariogenesis” OR “carious dentine” OR “carious teeth” OR “dental caries susceptibility” OR “dental decay” OR “dental fissure” OR “dental fissures fissure” OR “tooth root caries” OR “tooth caries tooth decay” OR “tooth fissure” OR “Decay Dental” OR “Carious Lesions” OR “Carious Lesion” OR “Lesion Carious” OR “Lesions Carious” OR “Carious Dentin” OR “Carious Dentins” OR “Dentin Carious” OR “Dentins Carious” OR “Dental White Spot” OR “Spot Dental White” OR “Spots Dental White” OR “White Spot Dental” OR “White Spots Dental” OR “Dental White Spots” OR “human caries”) AND TITLE-ABS-KEY (“Vaccine” OR “vaccines” OR “combined vaccine” OR “vaccin” OR “vaccine control” OR “vaccine efficacy” OR “vaccine potency” OR “vaccine safety” OR “vaccines combined”).

### 2.4. Bibliometric Indicators

The metadata found were exported to the database extracted to SciVal in CSV format; these were recorded in Microsoft Excel spreadsheets and analyzed by Institution, Author, Scopus Source, CiteScore quartile, and Collaboration indicators using tables. The present data collection and analysis were performed on September 18, 2021. Bibliometric indicators of production were presented, which refer to the number of documents and citations for universities, authors, and journals that publish scientific publications on caries vaccines. Likewise, collaboration indicators were analyzed, which were divided into national, international, and institutional collaboration, and single authorship of the scientific publications was analyzed. Finally, impact indicators were also analyzed, which were measured according to the quartiles of the scientific journals analyzed.

### 2.5. Data Analysis

In the present publication, various concepts were used for metric and citation analysis, which will be defined in summary form. The first indicator was the H index (h-index), a tool to determine the impact of the number of citations of a published scientific or academic manuscript, as a measure of the productivity of a university, a country, or a group of scientists [16, 17]. Similarly, the Source Normalized Impact per Paper (SNIP) was used, which is defined as the ratio of the number of journal citations per article and the potential of these in the thematic field and aims to elaborate a direct comparison of sources in different fields of evaluation [18]. The CiteScore was also an indicator used, which measures the average number of citations received per manuscript published in a given series and is calculated by taking as a reference the number of citations for all publications in the current year to the three previous years, classifying the highest values as the most relevant and impact, divided into four similar parts, each equivalent to a corresponding quartile [19]. Similarly, the Field-Weighted Citation Impact (FWCI) was calculated, an indicator from SciVal, which measures the impact of citations by comparing the actual number of citations obtained in a publication with the expected number of citations for manuscripts of the same type and is interpreted according to the result being greater or less than 1; if it is greater than 1, it denotes that the result takes more citations than the expected average; if it is less, it indicates the opposite [20, 21]. The SCImago Journal Rank (SJR) indicator was also evaluated, which is a metric used to evaluate the quality of scientific journals in the Scopus database [22] and is calculated by a repetitive process of “prestige” obtained by the manuscript through the other publications belonging to the network by the number of citations [23]. Similarly, Scholarly Output was used, an indicator that provides the number of manuscripts published by the institution and/or author to be evaluated in the journals indexed in Scopus. Finally, Citations per Publication, a metric that provides an average of the number of citations of each of the scientific manuscripts, Citation Count, which shows the total number of citations received by the bibliometric indicator to be evaluated up to the date of the last data cut-off, and Document Count, which shows the number of publications that an author, journal, university, or country has indexed in Scopus, were used. Subsequently, the bibliometric networks were elaborated to identify the different research metrics worldwide, using the VOSviewer software (version 1.6.10). This software is a tool used to visualize collaborative networks between different authors, journals, and institutions by identifying cocitation, co-occurrence, and coauthorship.

## 3. Results

### 3.1. Top 10 Universities with the Highest Scientific Production

Table 1 shows the ten universities with the highest scientific productivity on the development of vaccines against dental caries. Wuhan University (China) is positioned in the number one position as the institution with the highest scientific production on the subject, with 69 citations and 17.3 citations per article; it also becomes the institution with the highest impact. In the next two positions are the universities of Denmark (University of Aarhus and University of Copenhagen) with 27 citations and 27 citations per publication.

### 3.2. Top 10 Most Productive Authors

According to Table 2, Yan Huimin, Yang Jingyi, Zhou Dihan, Yang Yi, Li Yuhong, and Fan Mingwen top the list of most productive authors on the development of vaccines against dental caries, each with 3 papers produced on the subject. These researchers are also the most cited, the first four with 68 citations and 22.7 citations per publication and the last two with 55 citations and 18.3 citations per publication each.

### 3.3. Top 10 Most Productive Journals

The top ten journals with the highest number of publications on the development of vaccines against dental caries are shown in Table 3. The first two places being for Journal of Dental Research and Molecular Oral with 7 papers and 2 papers, respectively. However, only the first journal remains as one of the most cited journals (89 citations; 12.7 citations per publication), followed by European Journal of Oral Sciences (27 citations; 27 citations per publication) and BMC Oral Health (16 citations; 16 citations per publication) in the order mentioned, despite both having 1 paper published on the topic.

### 3.4. Number and Impact of Publications by Journal Quartile

Table 4 shows the number of papers conducted on the development of vaccines against dental caries, by the quartile of the journal where they were published. The largest number of papers (61%) is in *Q*1 (top 25%), 15% (*n* = 3) in *Q*2, 20% (*n* = 4) in *Q*3, and only 5% are in *Q*4.

### 3.5. Type of Collaboration

The collaboration pattern of the scientific output on dental caries vaccine development is shown in Table 5. The majority had national collaboration (*n* = 29), followed by institutional collaboration (*n* = 28) and to a lesser extent, international collaboration (*n* = 12) and single authorship (*n* = 4). With respect to citation rates and the impact demonstrated by this, it was obtained that national collaboration (968 citations; 33.4 citations per publication) exceeds international collaboration (374 citations; 31.2 citations per publication), institutional-only collaboration (263 citations; 9.4 citations per publication), and single authorship (3 citations; 0.8 citations per publication).

### 3.6. Collaborative Networks

**Figure 1:**
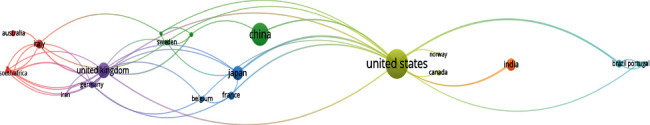
Coauthorship by country.

**Figure 2:**
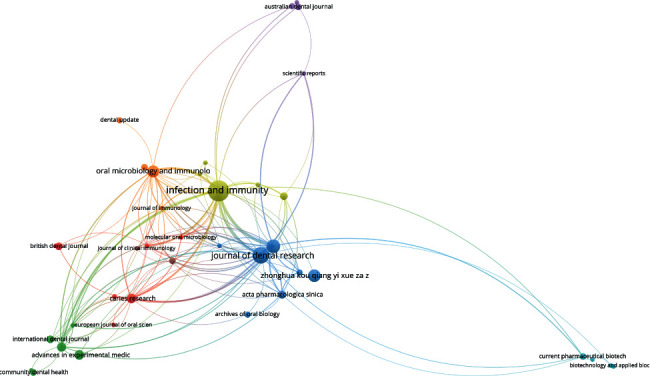
Citation by journal.

On analyzing coauthorship by country, with a minimum of 3 manuscripts per country, 3 large clusters were identified, corresponding to the United States, China, and United Kingdom, which condensed the largest number of coauthorships per country in relation to worldwide scientific production on dental caries vaccines (Figure 1). On the other hand, with a minimum of 2 manuscripts per journal, the Journal of Dental Research and Infection and Immunity were the ones that condensed the highest citation per journal since they were the two largest clusters (Figure 2).

### 3.7. Synthetic Knowledge Synthesis

**Figure 3:**
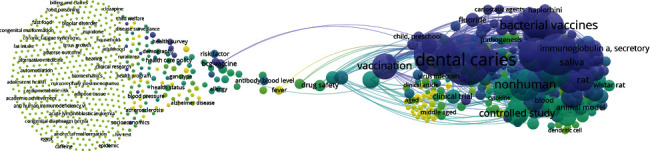
Synthetic knowledge synthesis.

When analyzing the co-occurrence of the index key words, the results of the synthetic knowledge synthesis revealed 5 major clusters corresponding to the topics “Dental caries,” “Bacterial vaccines,” “Immunoglobulin a secretory,” “Vaccination,” and “Saliva,” which have been researched since 2000 and are interrelated with other topics through the “Dental caries,” “Bacterial vaccines,” and “Saliva.” “Immunoglobulin a secretory,” “Vaccination,” and “Saliva” have been investigated since 2000 and are interrelated with other topics through 3 smaller clusters such as “Drug safety,” “Antibody blood level,” and “BCG vaccine” between 2005 and 2010 (Figure 3).

## 4. Discussion

As has been previously determined, because dental caries is a prevalent disease worldwide [24, 25] and can appear at any time of life [26, 27], the development of a vaccine would be an ideal method of prevention to reduce the impact of this pathology, even with the uncertainty and lack of scientific agreement found on the subject, it would limit the population from various complications present in this pathology, both medical and social [28]. The importance of the present study lies in providing an overview of the production in the scientific field, observing quantitatively, and prioritizing its relevance by promoting future research. For this reason, the main objective is to perform a bibliometric analysis of the global scientific production on the development of vaccines for dental caries, with a search based on the manuscripts present in the Scopus database, carried out until the year 2020.

The methodology used in the branch of bibliometrics deals with the use of quantitative tools for the evaluation of scientific production corresponding to indexed databases, using various metric indicators of production, collaboration, and impact [29]. The objective is to perform a distribution by dividing the elements to be evaluated (journals, keywords, authors, countries, and documents) into different groups to visualize the visual representation of the classification obtained [30]. Bibliometric studies are important because they have the advantage of being able to introduce a review process with systematic, reproducible, and transparent analysis, resulting in an improvement in the quality and prestige of the reviews. In addition, this type of manuscript is useful at the time of the observation of the literature, even before the beginning of the reading, manifesting itself as orientation of the researcher towards the publications with more influence in the investigated field, mapping without risks of existence of subjective biases [31]. Additionally, publications regarding this type of studies have become important in medicine, presenting an ascending production curve in the last 10 years, corresponding to more than 17 areas of relation, with dentistry being present in the top 15 with more than 40 indexed articles and 400 citations of 72.979 publications [32]. This is due to the growing popularity, uncomplicated acquisition, and use and analysis of bibliometric software and databases, facilitating the evaluation of a large amount of data, an aspect that other types of studies do not present [33]. In comparison with meta-analysis studies and systematic reviews, bibliometric studies have an advantage since it is possible to apply them in a scenario of heterogeneous results, in a homogeneous one but with low quantity results, when high quality publications are minimal and can cover quite wide review scopes, circumstances that are impossible to apply in meta-analytic and systematic studies [34].

In relation to the comparison of the results of the present bibliometric study, it was found that the Journal of Dental Research was the most productive and the most cited in the last decade with respect to the production of articles related to the development of vaccines against dental caries, a result similar to that found in another bibliometric study that analyzed the impact of articles related to the topic of dental caries in children, where it was positioned in the top 3 [35]. It was also positioned in the first place among the most cited journals in two bibliometric studies, one of which analyzed the most cited articles related to dental caries [36] and the other related to the most cited articles in dentistry [37]. Also, in a bibliometric study that analyzed the most cited articles in pediatric dentistry, the journal was positioned in the top 8 [38]. In addition, in a recent study that analyzed the research interest in silver diamine fluoride in dentistry, related to the prevention of dental caries, the journal ranked third, with an h-index of 15, which positioned it as the journal with the highest impact [39]. A bibliometric study related to the production of articles on vaccines has also been developed, in which the most productive journal was the New England Journal of Medicine and The Lancet. Although the results identified in the present bibliometric study are similar to the aforementioned studies, in some, the journal was not positioned in the first place as the most productive or the one with the highest impact. This may be due to the use of a different database than the one used in this study, such as Web of Science, and a larger number of documents and also because there was no restriction with respect to the year of publication of the document or any specification in the topic of vaccines or caries and studies of the development of these topics in multiple fields were analyzed, not only dentistry and in a more general manner [40]. The journals in quartile 1 were those that predominated, a result that agrees with another bibliometric study related to the topic [35]. The bibliometric studies show within their results a list of the 100 most cited authors, where Featherstone J.D.B. ranked first [36], in contrast to the present manuscript, where Yan Huimin, Yang Jingyi, Zhou Dihan, and Yang Yi ranked first as the most productive and cited. This is probably due to the use of a larger number of documents, since databases other than Scopus were analyzed and the range of years of analysis was wider, together with a broader analysis of the topic of dental caries in a more general way, unlike the present study which was more focused on vaccination against this pathology.

Similarly, in other bibliometric studies related to the topic of caries or caries prevention, similar findings were found [35–38], and this can be explained in part for the same reasons mentioned above. With respect to the most productive universities, the top 100 most cited articles on dental caries in children were analyzed, resulting in the University of Queensland in Australia, which had the highest number of articles affiliated to the list [35]. This is in contrast to the results identified in the present study, in which Wuhan University was the most productive and the most cited. This difference is because the first study mentioned was conducted over a longer time range, analyzing metadata developed from 1950 to 2019. Additionally, it was an analysis of articles on caries developed in early childhood, thus having a narrower age limit. A similar result was reported in another bibliometric study that analyzed the most cited articles in pediatric dentistry, with the University of Michigan being the most active [38]; this would be because metadata were analyzed from 1967 to 2013 using the Web of Science database, which would be a different range of years and database than ours. Finally, the type of national collaboration was predominant in our study, unlike another bibliometric study in which international collaboration corresponded to most articles. An underlying explanation is the fact that a different database was analyzed, in this case, Web of Science [39].

Regarding the limitations, the first was the number of manuscripts found in accordance with the topic, which is 122 publications, of which only 106 received bibliometric analysis. This is linked to another limitation, the analysis of the last 10 years, due to a certain limitation of the software used, including only publications from the period 2011–2020, which represent only 23% of all the available documents in accordance with the topic in Scopus. Third, the study was restricted to the analysis with only Scopus database, which may cause the loss of some important results of publications from nonindexed journals and may not fully reflect the complete research regarding the vaccine against dental caries. As a fourth limitation, there was a complex search for a consultant, as this person had to be specialized in bibliometrics, for the best results of the study, and to have access available in SciVal, due to the high-cost membership that this platform presents. The subsequent limitation was the limited measurement of impact in the manuscripts, since it is not possible to recognize with certainty whether the article to be analyzed has made a real contribution to the subject, limiting the study to the existence of citations in other articles. Besides, it should be considered that the search strategy, more specifically the formula, is perfectible over time, even if it has been elaborated in an advanced and specialized way, which could result in a bias of results in the future. Finally, in this study, the bibliographic search was performed using only the Scopus database, so it is recommended that future studies complement the scientometric analysis by including other databases [40–43].

However, strengths have also been found in this bibliometric study. The first of these is the relevance of being a pioneer in the subject in relation to the type of study, serving as an incentive base to carry out, strengthen, improve, and raise awareness in the scientific community about future research on the vaccine against dental caries. The next is regarding the use of Scopus as the only database for bibliometric analysis, which guarantees highly relevant results by presenting specific characteristics regarding its selectivity in the inclusion of journals, which must meet a high standard of research category, going through a rigorous peer review process. The main strength of this study was that it allowed us to identify, using bibliometric indicators, that research on the potential vaccine against dental caries can directly benefit human beings, although it is still a developing topic. In addition, it became evident that there are several countries and researchers who can benefit directly from this study because it would promote a potential collaborative network to join efforts to consummate the development of this vaccine. Finally, the results obtained in the study allow us to conclude that there should be a complement with research to be carried out in different indexed databases, such as Medline, SciELO, Embase, and Web of Science (WOS), with the objective of establishing in a more adequate way the impact and production according to the subject of the vaccine against dental caries.

## 5. Conclusions

In conclusion, most manuscripts regarding the development of dental caries vaccines were published in China and in Q1 quartile journals. In addition, Yan Huimin, Yang Jingyi, Zhou Dihan, Yang Yi, Li Yuhong, and Fan Mingwen were found to top the list of most productive authors. The Journal of Dental Research was also identified as the most productive and most cited journal.

## Figures and Tables

**Table 1:** Top 10 universities of scientific output in Scopus of all publication types about caries vaccine.

Institution	Country	Scholarly Output	Citations	Authors	Citations per Publication	Field-Weighted Citation Impact
Wuhan University	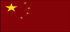	4	69	42	17.3	1.9
University of Indonesia	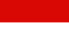	1	7	5	7	1
Aarhus University	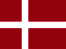	1	27	1	27	4.7
University of Copenhagen	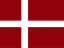	1	27	1	27	4.7
University of Glasgow	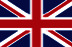	1	6	6	6	0.4
University of Leeds	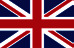	1	2	2	2	0.9
University of Zagreb	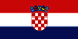	1	1	1	1	0.1
University of Amsterdam	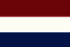	1	6	2	6	0.4
Vrije Universiteit Amsterdam	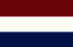	1	6	2	6	0.4
University of Porto	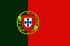	1	5	6	5	0.6

**Table 2:** Top 10 authors of scientific output in Scopus of all publication types about caries vaccine.

Name	Scholarly Output	Most recent publication	Citations	Citations per Publication	Field-Weighted Citation Impact	h-index
Yan Huimin	3	2015	68	22.7	2.4	20
Yang Jingyi	3	2015	68	22.7	2.4	14
Zhou Dihan	3	2015	68	22.7	2.4	13
Yang Yi	3	2015	68	22.7	2.4	10
Li Yuhong	3	2019	55	18.3	1.9	11
Fan Mingwen	3	2019	55	18.3	1.9	37
Manwar Narendra U.	2	2015	1	0.5	0	3
Yu Jie	2	2015	46	23.0	2.4	8
He Benxia	2	2012	54	27.0	2.8	8
Zhong Maohua	2	2015	46	23.0	2.4	13

**Table 3:** Top 10 of all publication types of caries vaccine by Scopus source.

Scopus source	Publications	Citations	Authors	Citations per Publication	Source Normalized Impact per Paper (SNIP)	CiteScore 2020	SCImago Journal Rank (SJR)
Journal of Dental Research	7	89	38	12.7	2.1	9.9	1.9
Molecular Oral Microbiology	2	11	12	5.5	1.1	6.7	1.1
Acta Stomatologica Croatica	1	1	1	1	0.7	1.4	0.3
Journal of the American Dental Association	1	1	2	1	1.7	4.2	0.5
BMC Oral Health	1	16	4	16	1.6	3.2	0.8
Community Dental Health	1	2	1	2	0.6	1.5	0.4
Archives of Oral Biology	1	1	5	1	0.9	3.9	0.7
European Journal of Oral Sciences	1	27	1	27	1.1	3.8	0.8
International Dental Journal	1	0	1	0	1.5	3.7	0.8
International Journal of Clinical Dentistry	1	0	5	0	0.5	0.1	0.1

**Table 4:** Publications by journal quartile by CiteScore percentile of all types of caries vaccine publications.

CiteScore quartile	2011	2012	2013	2014	2015	2016	2017	2018	2019	2020	Overall
Q1 (top 25%)	3	3	1	0	1	1	0	1	1	1	12
Q2 (top 26%–50%)	1	0	0	1	0	0	0	0	0	1	3
Q3 (top 51%–75%)	0	0	0	0	1	1	0	0	0	2	4
Q4 (top 76%–100%)	0	0	1	0	0	0	0	0	0	0	1
Total	4	3	2	1	2	2	0	1	1	4	20

**Table 5:** All publication types in caries vaccine by the amount of international, national, and institutional collaboration.

Metric	%	Scholarly Output	Citations	Citations per Publication	Field-Weighted Citation Impact
International collaboration	16.4	12	374	31.2	2.3
Only national collaboration	39.7	29	968	33.4	1.1
Only institutional collaboration	38.4	28	263	9.4	1.1
Single authorship (no collaboration)	5.5	4	3	0.8	0.4

## Data Availability

The data used in the statistical analysis of this study will be available upon authorization of the corresponding author.

## References

[1] Sillet A. (2013). Definition and use of bibliometrics in research. *Soins*.

[2] Iftikhar P. M., Ali F., Faisaluddin M., Khayyat A., De Gouvia De Sa M., Rao T. (2019). A bibliometric analysis of the top 30 most-cited articles in gestational diabetes mellitus literature (1946–2019). *Cureus*.

[3] Shuaib W., Khan M. S., Shahid H., Valdes E. A., Alweis R. (2015). Bibliometric analysis of the top 100 cited cardiovascular articles. *The American Journal of Cardiology*.

[4] Pulgar R., Jiménez-Fernández I., Jiménez-Contreras E., Torres-Salinas D., Lucena-Martín C. (2013). Trends in world dental research: an overview of the last three decades using the Web of science. *Clinical Oral Investigations*.

[5] Mayta-Tovalino F., Pacheco-Mendoza J., Diaz-Soriano A., Perez-Vargas F., Munive-Degregori A., Luza S. (2021). Bibliometric study of the national scientific production of all peruvian schools of dentistry in scopus. *International Journal of Dentistry*.

[6] GBD 2017 Disease and Injury Incidence and Prevalence Collaborators (2018). Global, regional, and national incidence, prevalence, and years lived with disability for 354 diseases and injuries for 195 countries and territories, 1990-2017: a systematic analysis for the Global Burden of Disease Study 2017. *Lancet*.

[7] Sabogal Á., Asencios J., Robles A. (2019). Epidemiological profile of the pathologies of the oral cavity in a Peruvian population: a 9-year retrospective study of 18,639 patients. *The Scientific World Journal*.

[8] Marsh P. D. (2009). Dental plaque as a biofilm: the significance of pH in health and caries. *Compendium of Continuing Education in Dentistry*.

[9] Maguire A., Clarkson J. E., Douglas G. V. (2020). Best-practice prevention alone or with conventional or biological caries management for 3- to 7-year-olds: the FICTION three-arm RCT. *Health Technology Assessment*.

[10] Lemos J. A., Palmer S. R., Zeng L. (2019). The biology of streptococcus mutans. *Gram-Positive Pathogens*.

[11] Bi Y., Xu Q., Su L. (2019). The combinations chitosan-pam (3) CSK (4) and chitosan-monophosphoryl lipid A: promising immune-enhancing adjuvants for anticaries vaccine PAc. *Infection and Immunity*.

[12] Yang H., Yan Z., Zhang Z., Realivazquez A., Ma B., Liu Y. (2019). Anti-caries vaccine based on clinical cold-adapted influenza vaccine: a promising alternative for scientific and public-health protection against dental caries. *Medical Hypotheses*.

[13] Kt S., Kmk M., Jimson N. B. (2013). Dental caries vaccine - a possible option?. *Journal of Clinical and Diagnostic Research*.

[14] Cherukuri G., Veeramachaneni C., Rao G. V., Pacha V. B., Balla S. B. (2020). Insight into status of dental caries vaccination: a review. *Journal of Conservative Dentistry*.

[15] Qamar Z., Alturki O. Y., Aljarallah A. F., Zeeshan T. (2021). A bibliometric analysis of top 100 cited articles on dental caries during 2000–2019. *Mymensingh Medical Journal: MMJ*.

[16] Hirsch J. E., Buela-Casal G. (2014). The meaning of the h-index. *International Journal of Clinical and Health Psychology*.

[17] Costas R., Bordons M. (2007). The h-index: advantages, limitations and its relation with other bibliometric indicators at the micro level. *Journal of Informetrics*.

[18] Villaseñor-Almaraz M., Islas-Serrano J., Murata C., Roldan-Valadez E. (2019). Impact factor correlations with scimago journal rank, source normalized impact per paper, eigenfactor score, and the citescore in radiology, nuclear medicine and medical imaging journals. *La Radiologia Medica*.

[19] Okagbue H. I., Bishop S. A., Oguntunde P. E., Adamu P. I., Opanuga A. A., Akhmetshin E. M. (2019). Modified CiteScore metric for reducing the effect of self-citations. *Telkomnika (Telecommunication Computing Electronics and Control)*.

[20] Purkayastha A., Palmaro E., Falk-Krzesinski H. J., Baas J. (2019). Comparison of two article-level, field-independent citation metrics: field-weighted citation impact (FWCI) and relative citation ratio (RCR). *Journal of Informetrics*.

[21] Waltman L., van Eck N. J. (2015). Field-normalized citation impact indicators and the choice of an appropriate counting method. *Journal of Informetrics*.

[22] Falagas M. E., Kouranos V. D., Arencibia-Jorge R., Karageorgopoulos D. E. (2008). Comparison of SCImago journal rank indicator with journal impact factor. *The FASEB Journal*.

[23] García-Pachón E., Arencibia-Jorge R. (2014). A comparison of the impact factor and the SCImago journal rank index in respiratory system journals. *Archivos de Bronconeumología (English Edition)*.

[24] Farooqi F. A., Khabeer A., Moheet I. A., Khan S. Q., Farooq I., ArRejaie A. S. (2015). Prevalence of dental caries in primary and permanent teeth and its relation with tooth brushing habits among schoolchildren in Eastern Saudi Arabia. *Saudi Medical Journal*.

[25] Sampaio F. C., Bönecker M., Paiva S. M. (2021). Dental caries prevalence, prospects, and challenges for latin America and caribbean countries: a summary and final recommendations from a regional consensus. *Brazilian Oral Research*.

[26] Kale S., Kakodkar P., Shetiya S., Abdulkader R. (2020). Prevalence of dental caries among children aged 5-15 years from 9 countries in the Eastern Mediterranean Region: a meta-analysis. *Eastern Mediterranean Health Journal*.

[27] Kamberi B., Koçani F., Begzati A. (2016). Prevalence of dental caries in Kosovar adult population. *International Journal of Dentistry*.

[28] Opydo-Szymaczek J., Borysewicz-Lewicka M., Andrysiak K. (2021). Clinical consequences of dental caries, parents’ perception of child’s oral health and attitudes towards dental visits in a population of 7-year-old children. *International Journal of Environmental Research and Public Health*.

[29] De Dios J., Moya M., Mateos Hernández M. A. (1997). Bibliometric indicators: characteristics and limitations of the analysis of scientific activity. *Anales Espanoles de Pediatria*.

[30] Boyack K. W., Small H., Klavans R. (2013). Improving the accuracy of co-citation clustering using full text. *Journal of the American Society for Information Science and Technology*.

[31] Zupic I., Čater T. (2015). Bibliometric methods in management and organization. *Organizational Research Methods*.

[32] Kokol P., Blažun Vošner H., Završnik J. (2021). Application of bibliometrics in medicine: a historical bibliometrics analysis. *Health Information and Libraries Journal*.

[33] Mustak M., Salminen J., Plé L., Wirtz J. (2021). Artificial intelligence in marketing: topic modeling, scientometric analysis, and research agenda. *Journal of Business Research*.

[34] Donthu N., Kumar S., Mukherjee D., Pandey N., Lim W. M. (2021). How to conduct a bibliometric analysis: an overview and guidelines. *Journal of Business Research*.

[35] Patil S. S., Sarode S. C., Sarode G. S. (2020). A bibliometric analysis of the 100 most cited articles on early childhood caries. *International Journal of Paediatric Dentistry*.

[36] Baldiotti A. L. P., Amaral-Freitas G., Barcelos J. F. (2021). The top 100 most-cited papers in cariology: a bibliometric analysis. *Caries Research*.

[37] Asiri F. Y., Kruger E., Tennant M. (2021). The top 100 most cited articles published in dentistry: 2020 update. *Healthcare*.

[38] Garcovich D., Marques Martinez L., Adobes Martin M. (2020). Citation classics in paediatric dentistry: a bibliometric study on the 100 most-cited articles. *European Archives of Paediatric Dentistry*.

[39] Jiang C. M., Duangthip D., Chan A. K. Y., Tamrakar M., Lo E. C. M., Chu C. H. (2021). Global research interest regarding silver diamine fluoride in dentistry: a bibliometric analysis. *Journal of Dentistry*.

[40] Quincho-Lopez A., Pacheco-Mendoza J. (2021). Research trends and collaboration patterns on polymyxin resistance: a bibliometric analysis (2010–2019). *Frontiers in Pharmacology*.

[41] Golpinar M., Demir E. (2020). Global research output of the cerebellum: yesterday, today, and tomorrow. *Journal of the Anatomical Society of India*.

[42] Demir E., Akmeşe Ö. F., Erbay H., Taylan-Özkan A., Mumcuoğlu K. Y. (2020). Bibliometric analysis of publications on house dust mites during 1980–2018. *Allergologia et Immunopathologia*.

[43] Kiraz S., Demir E. (2021). Global scientific outputs of schizophrenia publications from 1975 to 2020: a bibliometric analysis. *Psychiatric Quarterly*.

